# Dynamic Optimization Method for Broadband ADCP Waveform with Environment Constraints

**DOI:** 10.3390/s21113768

**Published:** 2021-05-28

**Authors:** Yongshou Yang, Shiliang Fang

**Affiliations:** Key Laboratory of Underwater Acoustic Signal Processing, Ministry of Education, Southeast University, Nanjing 210096, China; ysyang@seu.edu.cn

**Keywords:** waveform optimization, phase encoding, ambiguity function, nonlinear programming, ADCP

## Abstract

Broadband acoustic Doppler current profiler (ADCP) is widely used in agricultural water resource explorations, such as river discharge monitoring and flood warning. Improving the velocity estimation accuracy of broadband ADCP by adjusting the waveform parameters of a phase-encoded signal will reduce the velocity measurement range and water stratification accuracy, while the promotion of stratification accuracy will degrade the velocity estimation accuracy. In order to minimize the impact of these two problems on the measurement results, the ADCP waveform optimization problem that satisfies the environment constraints while keeping high velocity estimation accuracy or stratification accuracy is studied. Firstly, the relationship between velocity or distance estimation accuracy and signal waveform parameters is studied by using an ambiguity function. Secondly, the constraints of current velocity range, velocity distribution and other environmental characteristics on the waveform parameters are studied. For two common measurement applications, two dynamic configuration methods of waveform parameters with environmental adaptability and optimal velocity estimation accuracy or stratification accuracy are proposed based on the nonlinear programming principle. Experimental results show that compared with the existing methods, the velocity estimation accuracy of the proposed method is improved by more than 50%, and the stratification accuracy is improved by more than 22%.

## 1. Introduction

Acoustic Doppler current profiler (ADCP) is a popular Doppler sonar used for measuring water velocity and profile discharge in natural and human-made waterways. Due to the advantages of high estimation accuracy, multiple measurement parameters and low measurement cost, the ADCP has been widely used in the fields of hydrological survey, water resources exploration, underwater protection and navigation [1,2,3,4,5], etc. For example, 98% of United States Geological Survey (USGS) non-wading river discharge measurements were performed by ADCP in 2013 [6]. ADCP divides the river section into several vertical subsections. The average velocity and discharge of each subsection are obtained by measuring the velocity of multiple vertical layers in the subsection. Then, the discharge of all subsections is superimposed to get the discharge of the entire river section [7]. Therefore, high-precision velocity measurement is the cornerstone of accurate section discharge measurement. The following is a brief introduction to the concept and importance of stratification or layering. Stratification refers to dividing the entire echo into several segments during signal processing. Each segment is the echo generated by the scatterers in the corresponding depth water layer being illuminated by the transmitted sound wave. The velocity and discharge of each layer are estimated separately. Since the current velocity changes with depth, stratification can improve the accuracy of velocity and discharge measurement of entire river section and obtain the relationship between velocity and depth. The actual achievable layer thickness is generally determined by the transmitted pulse width of ADCP. The thinner the layer, the more details about the current velocity change with depth can be obtained, which is very important for many hydrological survey applications.

The measurement methods used in ADCP include pulse incoherent method [8], pulse coherent method [9,10,11] and broadband method [12,13,14]. Since the broadband method combines the advantages of the incoherent method and the coherent method, it has become the most popular choice. The accuracy of velocity estimation directly determines the performance of velocity distribution estimation, profile discharge estimation, underwater navigation and other application requirements, while the stratification accuracy determines the observation scale of velocity variation with depth. Therefore, the velocity estimation accuracy and water stratification accuracy are the core performance parameters of ADCP. The broadband method usually uses a phase-encoded pulse as the transmitted signal, its velocity estimation accuracy and water stratification accuracy are determined by the pulse duration and the average time of phase difference, respectively.

The research on the performance and uncertainty of velocity estimation has always been the focus in the field of acoustic Doppler current measurement. In many studies, the measurement uncertainty guidelines [15], Monte Carlo simulation [16] and American Aerospace Society [17] methods were used to analyze the uncertainty of various types of ADCP measurements strictly. Aurélien Despax et al. studied the uncertainty of ADCP discharge measurement caused by cross-section selection and human operation by using repeated measures experiments and put forward some strategies to reduce the measurement uncertainty [18]. ADCP is usually installed on a moving carrier to complete the measurement. References [19,20] proposed a method to improve the velocity estimation accuracy by compensating the carrier motion. The bias error in moving-carrier ADCP current measurement can be separated into two classes: calibration error and application error; the major sources of calibration error and the influence of parameters on the discharge uncertainty were analyzed in Reference [21]. It is an efficient method to analyze and verify the estimation performance of ADCP through laboratory calibration system, which can simulate a variety of different measurement application environments in a short time [22]. At the same time, analyzing the estimation performance and error sources of ADCP through field comparison is also an important performance research method, especially for those new principle-based instruments [23]. Reference [24] uses fractional Fourier transform to separate the component of strong scatterers from ADCP echo, which improves the accuracy and stability of water velocity estimation. ADCP is widely used in current measurement, and there are many ways to improve the performance of velocity estimation, but there are few studies related to the optimization of transmitted waveform. Reference [25] points out that the measurement deviation of the phase-encoded signal is mainly determined by the energy of the autocorrelation function of a single baseband pulse (SBPAF) and gives the calculation method of the coding phase that makes the SBPAF energy maximum. Although this method can reduce the signal spectrum broadening caused by phase discontinuity, it also increases the complexity of waveform design, hardware implementation and signal processing. Reference [26] deduces the Cramer Rao bound of broadband Doppler sonar, points out that the factors affecting the accuracy of single beam velocity estimation are divided into SNR and echo randomness and gives the qualitative selection strategy of waveform parameters. Reference [27] proposes a waveform design method of dual band coherent phase-encoded transmitted signal by dividing the signal spectrum into two. This method enriches the form of the transmitted signal of ADCP, but its estimation performance is still limited by the lack of coherent method, and this method does not consider the adaptation to the application environment. To sum up, it is a relatively efficient strategy to adjust the velocity estimation accuracy and stratification accuracy by changing the code length and number of code repeats (hereinafter referred to as the number of repeats), and this strategy is easy to adapt to the application environment. However, improving the velocity estimation accuracy will reduce the velocity measurement range and water stratification accuracy, and improving the stratification accuracy will also degrade the velocity estimation accuracy. The application environment of acoustic Doppler measurement is complex and diverse [28,29,30]. Due to the lack of specific strategies to adjust the waveform parameters according to the characteristics of the application environment, it is difficult to optimize the measurement performance of ADCP. In order to solve this problem, this paper introduces the constraints of sonar application environment, and researches the environment-adaptable waveform optimization methods of broadband ADCP based on the ambiguity function and nonlinear programming method.

The constraints of application environment on the waveform parameters of broadband ADCP are mainly reflected in the following aspects. In theory, the longer the code length and the more repeats, the higher the velocity estimation accuracy. In practice, it is necessary to consider the limitation of current velocity range, stratification accuracy requirement and other factors, and it is not advisable to blindly increase the code length and number of repeats. The velocity measurement range of the broadband method is inversely proportional to the code length and chip width (limited by the bandwidth of the transducer and receiver, the chip width is generally fixedly set to the reciprocal of the system bandwidth) [14]. In order to prevent the maximum current velocity from exceeding the velocity measurement range, there is an upper limit on code length. The layer thickness of the broadband method is proportional to the product of code length and number of repeats. The upper limit of the layer thickness requirement determines the upper limit of the product. The velocity estimation accuracy of the broadband method is inversely proportional to code length and number of repeats [13]. The lower limit of the velocity estimation accuracy requirement determines the lower limit of the product of code length and number of repeats. In order to ensure the output signal-to-noise ratio (SNR) of the matched filter of the receiver and the peak sidelobe level (PSL) of the autocorrelation function of the coded signal, there is a lower limit for code length. Therefore, the waveform design of the broadband ADCP must consider the above constraints of application environment.

In order to solve the problem that the existing waveform design methods lack the pertinence of application of environment characteristics and are difficult to realize the optimization of estimation performance, this paper introduces the constraints of the application environment, and uses an ambiguity function and nonlinear programming method to design two dynamic configuration methods of waveform parameters with environmental adaptability and optimal estimation performance. Section 2 introduces the basic operating principle of broadband ADCP. Section 3 employs the ambiguity function tool to broadband ADCP for the first time, studies the relationship between the velocity estimation accuracy of phase-encoded signal and its waveform parameters by using the ambiguity function, and gives the performance cost functions of velocity estimation and stratification. Then, aiming at the measurement application that prioritizes high velocity estimation accuracy or high stratification accuracy, a velocity estimation accuracy priority (VEAP) or a water stratification accuracy priority (WSAP) waveform optimization method are proposed. The constraints of the current velocity range, velocity estimation accuracy requirements and stratification accuracy requirements on the waveform parameters of the phase-encoded signal are studied in detail. Both methods model the design task of optimal waveform as a nonlinear programming problem with multiple inequality constraints and obtain waveform parameters that satisfy the constraints of the application environment and make the velocity estimation accuracy or stratification accuracy optimal. In Section 4, a principal prototype was built using an onshore ADCP calibration system. The experimental results demonstrate the correctness and practicability of the waveform design methods proposed by this paper. Finally, Section 5 highlights the conclusions of this study.

## 2. Principle of Broadband ADCP

**Figure 1:**
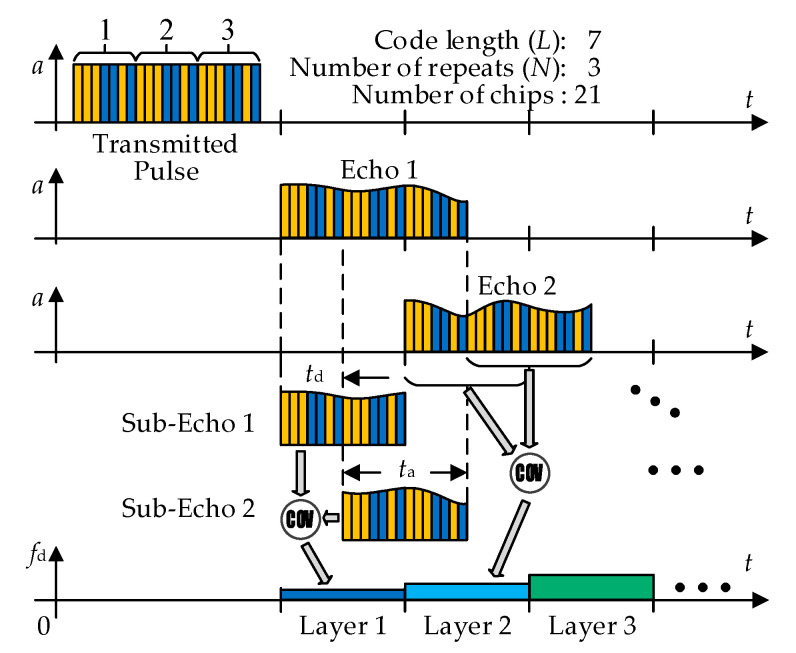
Velocity estimation principle of broadband Doppler sonar.

Broadband Doppler sonar usually uses a phase-encoded pulse as the transmitted signal for achieving higher range and velocity accuracy. The water profile is divided into multiple adjacent layers according to the length of the transmitted signal. First, the radial Doppler frequency shift of single beam in a single layer is estimated, and then the velocity profile in the geodetic coordinate system is obtained by combining the attitude sensing data and the radial Doppler frequency shift of multiple beams. The common velocity estimation method of broadband Doppler sonar is complex covariance algorithm, which estimates the average phase change of two sub-echoes in a single layer to get the radial Doppler shift of the layer. The time delay of the two sub-echoes is called as correlation delay, and the duration of the single sub-echo is named as average time of phase difference. The current velocity estimation principle of broadband Doppler sonar is shown in Figure 1, where the code length and number of repeats are set as 7 and 3, respectively. The code selection criterion in the broadband method is that the main autocorrelation peak should be as sharp as possible, and the secondary autocorrelation peak should be as low as possible. There are some popular codes, such as Barker codes, minimum peak sidelobe (MPS) codes and pseudo-random noise sequences.

In Figure 1, *t*_d_ represents the correlation delay, *t*_a_ is the average time of the phase difference and the calculation formula of water layer thickness (oblique thickness along the beam direction) Δ*z* is:

(1) $$\Delta z=\frac{c}{2}t_{a}$$

where *c* is the underwater sound velocity. When the total chip number of the transmitted pulse is fixed, the more symbols *t*_a_ contains, the smaller the standard deviation of velocity estimation [25]. Therefore, the *t*_a_ is usually configured equal to the time width of *N* − 1 repetitive coding and *t*_d_ is equal to the time width of single coding, namely:

(2) $$t_{a}=(N-1)L\tau ,\;t_{d}=L\tau$$

where *N* is the number of repeats, *L* is the code length and *τ* is the chip width. Substituting *t*_a_ in Equation (2) into (1), we get the following result:

(3) $$\Delta z=\frac{c}{2}(N-1)L\tau$$

The absolute value of velocity measurement range is called ambiguity velocity. In the broadband method, the ambiguity velocity is inversely proportional to the correlation delay. Assuming the phase change range of the two sub-echoes is [−π, π], the ambiguity velocity *v*_max_ is calculated as follows [13]:

(4) $$v_{\max}=\frac{\lambda }{2}f_{\mathrm{dmax}}=\frac{\lambda }{4t_{d}}$$

where *λ* is the wavelength and *f*_dmax_ is the maximum Doppler shift. According to the complex covariance algorithm [13], the Doppler angular shift can be calculated by the change rate of echo phase within the correlation delay *t*_d_, so the maximum Doppler angular frequency 2π*f*_dmax_ is equal to the ratio of the maximum phase change π that may occur within *t*_d_ to the time interval *t*_d_. Finally, *f*_dmax_ = 1/(2*t*_d_).

## 3. Methods

### 3.1. Relationship Between Velocity Estimation Accuracy and Waveform Parameters

The ambiguity function is a tool used in the radar field to analyze the effects of time delay and Doppler frequency shift on radar echoes. In view of the similarity between Doppler sonar and radar systems, it is also feasible to apply ambiguity functions to the field of sonar. By studying the ambiguity function of the transmitted waveform, the relationship between the waveform parameters and the resolution, ambiguity and estimation accuracy of the Doppler sonar can be determined. The definition of the complex ambiguity function is as follows:

(5) $$\chi (t,f)=\int_{-\infty }^{\infty }x(s)x^{*}(s-t)e^{j2\pi fs}ds$$

where *t* is the time delay relative to the expected matched filter peak output, *f* is the Doppler shift between the actual echo and the transmitted signal, *x*(*t*) is usually the complex envelope of the signal and the higher the value of *χ*(*t*, *f*), the stronger the resolution of the system on this (*t*, *f*). Ambiguity function is usually defined as the amplitude function of complex ambiguity function, namely |*χ*(*t*, *f*)|.

The ambiguity function of the phase-encoded signal is based on the ambiguity function of the rectangular pulse signal. It is assumed that the complex envelope of the rectangular pulse is defined as follows:

(6) $$x_{r}(t)=\{\begin{array}{l} 1/\sqrt{\tau_{r}},\;\left|t\right|\leq \tau_{r}/2\\ 0,\;\left|t\right|>\tau_{r}/2\; \end{array}$$

where *τ*_r_ is the pulse width and the amplitude is normalized. The expression of *x*_r_(*t*) complex ambiguity function is as follows [31]:

(7) $$\chi_{r}(t,f)=\frac{\tau_{r}-\left|t\right|}{\tau_{r}}\cdot \frac{\sin\pi (\tau_{r}-\left|t\right|)f}{\pi (\tau_{r}-\left|t\right|)f}e^{j\pi tf},\;\left|t\right|\leq \tau_{r}$$

Calculate the amplitude of the complex ambiguity function and regard the result as the ambiguity function expression of the rectangular pulse, which is shown in Equation (8):

(8) $$\left|\chi_{r}(t,f)\right|=\left(\frac{\tau_{r}-\left|t\right|}{\tau_{r}}\right)\left|\frac{\sin\pi (\tau_{r}-\left|t\right|)f}{\pi (\tau_{r}-\left|t\right|)f}\right|,\;\left|t\right|\leq \tau_{r}$$

The complex envelope of repeated binary phase-encoded signal can be expressed as:

(9) $$x(t)=\sum_{n=0}^{NL-1}x_{r}\left(t-n\tau \right)e^{j\theta_{n}}$$

where *θ_n_* is equal to 0 or π, represents the polarity of biphase code, *L* is code length, *τ* is chip width and *N* is the number of repeats. By substituting Equation (9) into the definition formula of complex ambiguity function, the following results are obtained:

(10) $$\begin{array}{l} \chi (t,f)=\sum_{m=0}^{NL-1}\sum_{n=0}^{NL-1}e^{j(\theta_{m}+\theta_{n})}\int_{-\infty }^{\infty }x_{r}(s-m\tau )x_{r}^{*}(s-t-n\tau )e^{j2\pi fs}ds\\ \;=\sum_{m=0}^{NL-1}e^{j2\pi fm\tau }\sum_{n=0}^{NL-1}e^{j(\theta_{m}+\theta_{n})}\chi_{r}(t-m\tau +n\tau ,f) \end{array}$$

where *χ*_r_(*t*, *f*) is the complex ambiguity function of the rectangular pulse, the expression is as Equation (7) and *τ*_r_ equals *τ*. Conducting double summation decomposition on Equation (10), the complex ambiguity function of *N* times repeated *L*-bit coded pulse is:

(11) $$\begin{array}{l} \chi (t,f)=\sum_{n=1-NL}^{0}\chi_{r}(t-n\tau ,f)\sum_{m=0}^{NL-\left|n\right|-1}e^{j2\pi m\tau f}e^{j\left(\theta_{m}+\theta_{m-n}\right)}\\ \;+\sum_{n=1}^{NL-1}e^{j2\pi n\tau f}\chi_{r}(t-n\tau ,f)\sum_{m=0}^{NL-\left|n\right|-1}e^{j2\pi m\tau f}e^{j\left(\theta_{m}+\theta_{m+n}\right)} \end{array}$$

Since the range of *χ*_r_(*t*, *f*) on the *t*-axis is |*t*| ≤ *τ*, *χ*_r_(*t*, *f*) with different delays in Equation (11) will not be aliased, and the ambiguity function |*χ*_r_(*t*, *f*)| is equal to the sum of the amplitudes of all the summation terms in Equation (11). Equation (11) shows that the ambiguity function of the phase-encoded signal is superimposed by rectangular pulse ambiguity functions with different delays after amplitude weighting, and the weighting coefficient is related to the autocorrelation characteristics of the encoding. The distance and speed resolution performance of |*χ*_r_(*t*, *f*)| is mainly determined by its main peak. Taking *n* = 0 in Equation (11) to obtain the expression of the ambiguity function in the main peak range is:

(12) $$\left|\chi \left(t,\;f\;\right)\right|=\left|\chi_{r}\left(t,\;f\;\right)\right|\cdot \left|\overset{NL-1}{\underset{m=0}{\sum }}e^{\;j2\pi m\tau f}\right|,\;\left|t\right|\text{<}\tau ,\left|\;f\;\right|\text{<}\frac{1}{\tau }\;=\frac{\tau -\left|t\right|}{\tau }\cdot \left|\frac{\sin[\pi (\tau -\left|t|)\;f\;\right]}{\pi (\tau -|t|)\;f}\right|\;\cdot \left|\frac{\sin(\pi NL\tau \;f\;)}{\sin(\pi \tau \;f\;)}\right|,\;\left|t\right|\text{<}\tau ,\left|\;f\;\right|\text{<}\frac{1}{\tau }$$

**Figure 2:**
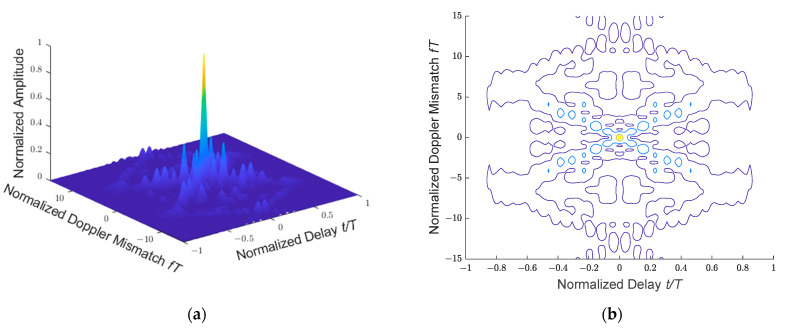
Ambiguity function of a 13-bit Barker code phase modulation signal: (**a**) ambiguity function of 3D perspective; (**b**) contour plot of ambiguity function in (**a**).

The ambiguity function of the 13-bit Barker code phase modulation signal is shown in Figure 2, and the contour plot of Figure 2a is shown in Figure 2b. Figure 2 is the graph of the square of the modulus of Equation (12) when *N* = 1 and *L* = 13. The amplitude, frequency shift and delay in the figure are all normalized, and *T* is the transmitted pulse width (*T* equals *NLτ*).

Based on the ambiguity function, the relationship between the velocity estimation accuracy and the waveform parameters such as code length and number of repeats can be concluded, and the cost function of velocity estimation performance can be estimated, which provides a theoretical basis for the optimal waveform design. Let *t* = 0 in Equation (12) to obtain the velocity ambiguity function of the phase-encoded signal:

(13) $$\left|\chi \left(0,\;f\;\right)\right|=\left|\frac{\sin(\pi NL\tau \;f\;)}{\pi \tau \;f\;}\right|,\left|\;f\;\right|\text{<}\frac{1}{\tau }$$

It can be seen from Equation (13) that the greater the product of code length and the number of repeats, the greater the PSL of the sin(π*NLτf*)/(π*τf*) structure, the more obvious the “pushpin” shape of the velocity ambiguity graph, and the less the influence of the multi-valued ambiguity on the velocity estimation results.

The accuracy of frequency estimation is determined by the second derivative of the velocity ambiguity function |*χ*(0, *f*)| [32], and the relational expression is shown in Equation (14). Equation (14) means the sharpness of the frequency dimension of the ambiguity function at point (0,0). The smaller the value of $\sigma_{f}^{2}$, the sharper the peak, and the higher the accuracy of velocity estimation. The velocity estimation accuracy can be greatly improved through reasonable configuration of the waveform parameters.

(14) $$\sigma_{f}^{2}=\frac{-1}{\frac{E}{E_{n}}{\left[\frac{\partial^{2}}{\partial f^{2}}\left|\chi \left(0,f\right)\right|\right]}_{f=0}}$$

where *E* is the energy of signal received by receiver, *E*_n_ is the noise energy per unit bandwidth of receiver, *E*_n_ = *kT*_k_*F*_n_, *k* is the Boltzmann constant (*k* = 1.38 × 10^−23^ J/K), *T*_k_ is the thermodynamic temperature corresponding to 17 °C and *F*_n_ is the noise coefficient of receiver. Substituting Equation (13) into (14), the following results are obtained:

(15) $$\sigma_{f}^{2}=\frac{3E_{n}}{E\pi^{2}N^{3}L^{3}\tau^{2}}$$

The standard deviation of velocity estimation is obtained by substituting the relationship between Doppler shift and velocity *v* = *λf*/2 into Equation (15):

(16) $$\sigma_{v}=\frac{\lambda }{2\pi }{\left[\frac{3}{(E/E_{n})N^{3}L^{3}\tau^{2}}\right]}^{1/2}$$

Equation (16) shows that the standard deviation of velocity estimation is inversely proportional to *N*^3/2^ (the 3/2 power of number of repeats), *L*^3/2^ (the 3/2 power of code length) and *τ* (the chip width). That is to say, the wider the chip, the longer the code, and the more repeats, the higher the accuracy of velocity estimation. In practice, the design of the sonar waveform needs to consider the actual current characteristics of measurement applications. In the following, specific phase-encoded waveform design methods are proposed for two common applications with high velocity estimation accuracy requirements and high-water stratification accuracy requirements.

### 3.2. Velocity Estimation Accuracy Priority (VEAP) Waveform Optimization Method

**Figure 3:**
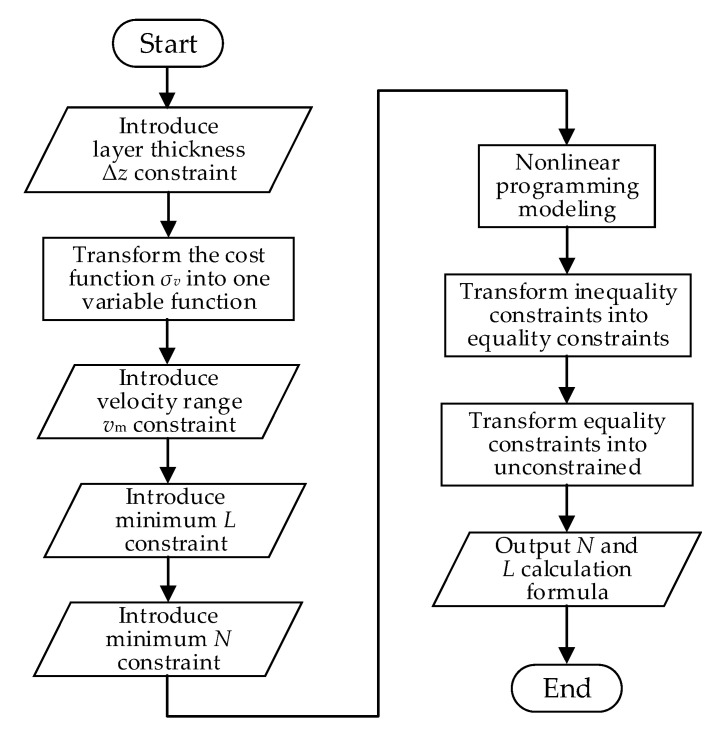
Flow chart of the VEAP method.

For the current measurement application that prioritizes high velocity estimation accuracy, priority is given to achieve high velocity accuracy. In this section, a configuration method of code length and number of repeats is proposed, which can satisfy the constraints of stratification accuracy and velocity estimation range and optimize the velocity estimation accuracy. From the analysis in Section 3.1, we can see that the best velocity estimation accuracy can be obtained when we maximize the code length, the number of repeats and chip width of the transmitted signal. Limited by the bandwidth of the transducer and receiver, the chip width is generally fixed as the inverse of the system bandwidth. Extending the code length will reduce the ambiguity velocity, which may cause the current velocity exceeds the velocity estimation range of the sonar; increasing the number of repeats will increase the layer thickness, resulting in a decrease in the ranging resolution. Therefore, while improving the velocity estimation accuracy, it is necessary to take into account the constraints, such as stratification accuracy requirement and velocity estimation range, and not blindly increase the code length and the number of repeats. It can be seen from the above analysis that the waveform design in this application is a nonlinear programming problem constrained by multiple inequalities. The solution is to first transform the cost function from two-variable function to a one-variable function, and then transform inequality constrained optimization into equality constrained optimization by introducing slack variables, and then introduce Lagrangian multipliers to transform equality constrained optimization into unconstrained optimization. The flow chart of the VEAP method is shown in Figure 3.

Based on the relationship between layer thickness and waveform parameters, the performance cost function of velocity estimation is transformed into a one-variable function. The calculation formula of the layer thickness Δ*z* in the broadband method is shown in Equation (3), assuming *g*(Δ*z*) = 2Δ*z*/(*cτ*), Equation (3) is equivalent to:

(17) L=g(Δz)/(N−1)

By substituting Equation (17) into (16), the cost function *σ_v_* is transformed into a single variable function:

(18) $$\sigma_{v}=\frac{\lambda }{2\pi }{\left[\frac{3}{\left(E/E_{n})g^{3}(\Delta z\right)}\right]}^{1/2}{\left(\frac{N-1}{N}\right)}^{3/2}$$

Equation (19) shows that the cost function and the number of repeats are non-linear. In addition, when *N* is fixed, the larger Δ*z*, the higher the accuracy of velocity estimation, so Δ*z* takes the maximum allowable layer thickness Δ*z*_m_.

The following is a detailed analysis of the constraints on the waveform parameters, such as current velocity range and stratification accuracy requirement. The current velocity range constraint makes *N* have a lower limit. The correlation delay is inversely proportional to the ambiguity velocity, and the maximum correlation delay must ensure that no velocity estimation ambiguity occurs, that is, the ambiguity velocity should be greater than the to be measured maximum current velocity. In addition, Doppler tolerance of the Barker code is poor [31]. In order to control the Doppler mismatch loss within 1dB, the phase change caused by the maximum current velocity generally does not exceed π/2, that is, the ambiguity velocity should be greater than 2 times the maximum current velocity to be measured. Thus, the inequality between maximum radial velocity *v*_m_ and correlation delay *t*_d_ is obtained:

(19) $$t_{d}\leq \frac{\lambda }{8v_{m}}$$

Substituting the *t*_d_ expression in Equation (2) into (19), we get the following result:

(20) $$L\leq \frac{\lambda }{8\tau v_{m}}=L_{\mathrm{mid}}$$

where *L*_mid_ = *λ*/(8*τv*_m_) represents the upper limit of the code length determined by the current velocity range. Combining Equations (17) and (20) to obtain a lower limit of *N*:

(21) $$N\geq \frac{16v_{m}\Delta z_{m}}{c\lambda }+1$$

The characteristics of the broadband method require that the number of repeats is not less than 2:

(22) N≥2

The lower limit of the PSL of code autocorrelation function makes *L* have a lower limit. Combining Equation (17) to obtain the constraint of the minimum allowable code length *L*_min_ on *N* is:

(23) $$N\leq \frac{2\Delta z_{m}}{c\tau L_{\min}}+1$$

Then, three slack variables $r_{1}^{2}$, $r_{2}^{2}$ and $r_{3}^{2}$ are introduced to transform inequality constraints into equality constraints. According to Equations (21)−(23) the transformation result is:

(24) $$\{\begin{array}{c} \frac{16v_{m}\Delta z_{m}}{c\lambda }+1-N+r_{1}^{2}=0\\ N-\frac{2\Delta z_{m}}{c\lambda L_{\min}}-1+r_{2}^{2}=0\\ 2-N+r_{3}^{2}=0 \end{array}$$

In order to transform the equality constrained optimization issue into the unconstrained optimization, Lagrangian multipliers *μ*_1_, *μ*_2_ and *μ*_3_ need to be introduced. According to Equation (24), the Lagrangian function *f*(*N*,$r_{1}^{2}$, $r_{2}^{2}$, $r_{3}^{2}$, *μ*_1_, *μ*_2_, *μ*_3_) is:

(25) $$\begin{array}{l} f(N,r_{1}^{2},r_{2}^{2},r_{3}^{2},\mu_{1},\mu_{2},\mu_{3})=\sigma_{v}\\ \;+\mu_{1}\left(\frac{16v_{m}\Delta z_{m}}{c\lambda }+1-N+r_{1}^{2}\right)\\ \;+\mu_{2}\left(N-\frac{2\Delta z_{m}}{c\lambda L_{\min}}-1+r_{2}^{2}\right)\\ \;+\mu_{3}\left(2-N+r_{3}^{2}\right) \end{array}$$

After calculating the partial derivative of the Lagrangian function with respect to the respective variables, an equation group after simplification is obtained:

(26) $$\{\begin{array}{r} \frac{\partial \sigma_{v}}{\partial N}-\mu_{1}+\mu_{2}-\mu_{3}=0\\ \mu_{1}\left(\frac{16v_{m}\Delta z_{m}}{c\lambda }+1-N\right)=0\\ \mu_{2}\left(N-\frac{2\Delta z_{m}}{c\lambda L_{\min}}-1\right)=0\\ \mu_{3}\left(2-N\right)=0\\ \mu_{1}\geq 0,\mu_{2}\geq 0,\mu_{3}\geq 0 \end{array}$$

where the expression of ∂*σ_v_*/∂*N* is:

(27) $$\frac{\partial \sigma_{v}}{\partial N}=\frac{3\lambda }{4\pi }{\left[\frac{3}{\left(E/E_{n})g^{3}(\Delta z_{m}\right)}\right]}^{1/2}{\left(\frac{N-1}{N^{5}}\right)}^{1/2}$$

**Figure 4:**
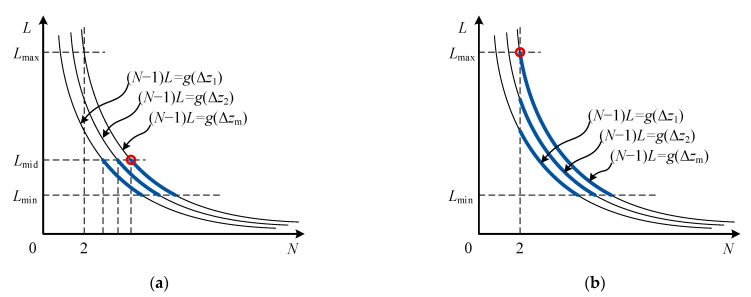
Feasible region of cost function of velocity estimation accuracy: (**a**) the case of *v*_m_Δ*z*_m_ ≥ *cλ*/16; (**b**) the case of *v*_m_Δ*z*_m_ < *cλ*/16.

According to the relationship between the expression on the right side of Equations (21) and (2), the solution of equation group (27) can be divided into two cases: *v*_m_Δ*z*_m_ ≥ *cλ*/16 and *v*_m_Δ*z*_m_ < *cλ*/16. When *v*_m_Δ*z*_m_ ≥ *cλ*/16, the value range of *N* is shown in Figure 4a. The thin solid lines in the figure represent curve cluster obtained by Equation (17), all Δ*z* with different values satisfies Δ*z* ≤ Δ*z*_m_. The thick solid line represents the feasible region of *N*. The cost function *σ_v_* takes the global optimal value at the red circle in Figure 4a. At this point, the expressions of *L* and *N* are:

(28) $$L=\left[\frac{\lambda }{8\tau v_{m}}\right],N=\left[\frac{g(\Delta z_{m})}{L}+1\right]=\left[\frac{16\Delta z_{m}v_{m}}{c\lambda }+1\right]$$

where the brackets indicate rounding. Equation (28) shows that when *v*_m_Δ*z*_m_ ≥ *cλ*/16, the standard deviation of velocity estimation can be minimized by first determining the code length by the velocity estimation range constraint and then determining the number of repeats by the stratification accuracy constraint. The minimum *σ_v_* obtained in this case is:

(29) $$\sigma_{v\min}=\frac{16\lambda }{\pi }{\left[\frac{3}{2(E/E_{n})}\right]}^{1/2}{\left(\frac{c\tau v_{m}}{16v_{m}\Delta z_{m}+c\lambda }\right)}^{3/2}$$

When *v*_m_Δ*z*_m_ < *cλ*/16, the feasible region of *N* is shown in Figure 4b. The cost function *σ_v_* takes the global optimal value at the black circle in Figure 4b. At this point, the expressions of *L* and *N* are:

(30) $$L=\left[g(\Delta z_{m})\right]=\left[\frac{2\Delta z_{m}}{c\tau }\right],N=2$$

where the square brackets indicate rounding. Equation (30) shows that when *v*_m_Δ*z*_m_ < *cλ*/16, the standard deviation of velocity estimation can be minimized by first setting the number of repeats to 2 and then determining code length by the stratification accuracy constraint. The minimum *σ_v_* obtained in this case is:

(31) $$\sigma_{v\min}=\frac{\lambda }{16\pi }{\left[\frac{3c^{3}\tau^{3}}{(E/E_{n}){\left(\Delta z_{m}\right)}^{3}}\right]}^{1/2}$$

In summary, the longest code length and the smallest number of repeats in the feasible region can minimize the velocity estimation standard deviation in the VEAP method.

### 3.3. Water Stratification Accuracy Priority (WSAP) Waveform Optimization Method

**Figure 5:**
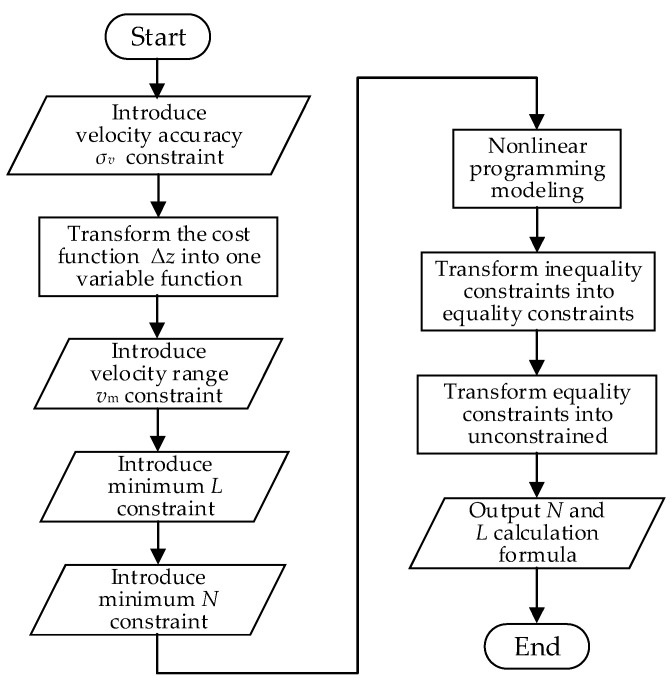
Flow chart of the WSAP method.

For the current measurement application that prioritizes high stratification accuracy, priority is given to achieve small layer thickness. In this section, a configuration method of code length and number of repeats is proposed, which can satisfy the constraints of velocity estimation accuracy and velocity estimation range and optimize the stratification accuracy. It can be seen from Equation (3) that the thinnest layer thickness can be obtained by selecting the smallest number of repeats, code length and chip width. Limited by the bandwidth of the transducer and receiver, the chip width is generally fixed as the inverse of the system bandwidth. Shortening the code length will increase the ambiguity velocity, and the phase change caused by the same velocity will decrease, which will lead to the greater influence of phase noise; thus reducing the number of repeats will reduce the time bandwidth product of the echo segment from which the phase difference is estimated, which will lead to the decline of velocity estimation accuracy. Therefore, when increasing the hierarchical accuracy, it is necessary to consider constraints such as velocity estimation accuracy, velocity estimation range, etc., and cannot reduce the number of code length and repeats. From the above analysis, it can be seen that the waveform design in this case is also a nonlinear programming problem with multiple inequality constraints, but the cost function changes into the Equation (3). The solution is similar to that in Section 3.2 and will not be repeated here. The flow chart of the WSAP method is shown in Figure 5.

Based on the relationship between velocity estimation accuracy and waveform parameters, the cost function of stratification accuracy is transformed into a one-variable function. Assuming that the maximum allowable velocity standard deviation is *σ_v_*_m_, the velocity estimation accuracy constraint can be expressed as:

(32) $$\sigma_{v}=\frac{\lambda }{2\pi \tau }{\left(\frac{3E_{n}}{EN^{3}L^{3}}\right)}^{1/2}\leq \sigma_{vm}$$

The Equation (32) is transformed into:

(33) $$L=y(\sigma_{v})/N$$

where *y*(*σ_v_*) is:

(34) $$y(\sigma_{v})={\left(\frac{3\lambda^{2}}{4\pi^{2}\tau^{2}\sigma_{v}^{2}(E/E_{n})}\right)}^{1/3}$$

By substituting Equation (33) into (3), the cost function Δ*z* is transformed into a single variable function:

(35) $$\Delta z=\frac{c\tau (N-1)}{2N}y(\sigma_{v})$$

Equation (35) shows that the cost function and the number of repeats are non-linear. In addition, when *N* is fixed, the larger *σ_v_*, the higher the stratification accuracy, so *σ_v_* takes the maximum allowable standard deviation *σ_v_*_m_.

The following is a detailed analysis of the constraints on the waveform parameters, such as current velocity range and velocity estimation accuracy requirement. The restriction of current velocity range on *N* has been studied in detail in Section 3.2, and only the final expression is given:

(36) $$N\geq \frac{8\tau v_{m}}{\lambda }y(\sigma_{v})$$

The characteristics of the broadband method require that the number of repeats is not less than 2:

(37) N≥2

The lower limit of the PSL of code autocorrelation function makes *L* have a lower limit. Combining Equation (32) to obtain the constraint of the minimum allowable code length *L*_min_ on *N* is:

(38) $$N\leq \frac{y(\sigma_{v})}{L_{\min}}$$

The method of transforming inequality constrained optimization into unconstrained optimization is similar to that in Section 3.2. The equations obtained by partial derivation of Lagrange function are given directly:

(39) $$\{\begin{array}{r} \frac{\partial \Delta z}{\partial N}-\mu_{1}+\mu_{2}-\mu_{3}=0\\ \mu_{1}\left(\frac{8\tau v_{m}}{\lambda }y(\sigma_{v})-N\right)=0\\ \mu_{2}\left(N-\frac{y(\sigma_{v})}{L_{\min}}\right)=0\\ \mu_{3}\left(2-N\right)=0\\ \mu_{1}\geq 0,\mu_{2}\geq 0,\mu_{3}\geq 0 \end{array}$$

where the expression of ∂Δ*z*/∂*N* is:

(40) $$\frac{\partial \Delta z}{\partial N}=\frac{c\tau y(\sigma_{v})}{2N^{2}}$$

**Figure 6:**
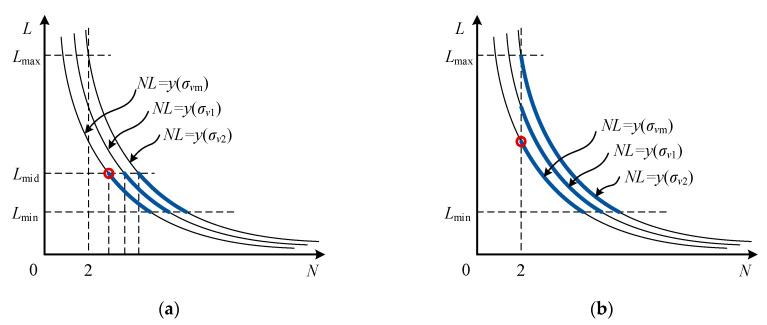
Feasible region of cost function of water stratification accuracy: (**a**) the case of *y*(*σ_v_*_m_) ≥ 2*L*_mid_; (**b**) the case of *y*(*σ_v_*_m_) < 2*L*_mid_.

According to the relationship between the expression on the right side of Equations (36) and (2), the solution of equation group (39) can be divided into two cases: *y*(*σ_v_*_m_) ≥ 2*L*_mid_ and *y*(*σ_v_*_m_) < 2*L*_mid_. When *y*(*σ_v_*_m_) ≥ 2*L*_mid_, the value range of *N* is shown in Figure 6a. The thin solid lines in the figure represent curve cluster obtained by Equation (33), all *σ_v_* with different values satisfies *σ_v_* ≤ *σ_v_*_m_. The thick solid line represents the feasible region of *N*. The cost function Δ*z* takes the global optimal value at the black circle in Figure 6a. At this point, the expressions of *L* and *N* are:

(41) $$L=\left[\frac{\lambda }{8\tau v_{m}}\right],\;N=\left[8v_{m}{\left(\frac{3\tau }{4\lambda \pi^{2}\sigma_{vm}^{2}(E/E_{n})}\right)}^{1/3}\right]$$

where the brackets indicate rounding. Equation (41) shows that when *y*(*σ_v_*_m_) ≥ 2*L*_mid_, the layer thickness can be minimized by first determining the code length by the velocity estimation range constraint and then determining the number of repeats by the velocity estimation accuracy constraint. The minimum layer thickness Δ*z*_min_ obtained in this case is:

(42) $$\Delta z_{\min}={\left(\frac{3c^{2}\lambda^{2}}{32\pi^{4}\tau \sigma_{vm}^{2}(E/E_{n})}\right)}^{1/3}-\frac{c\lambda }{16v_{m}}$$

When *y*(*σ_v_*_m_) < 2*L*_mid_, the feasible region of *N* is shown in Figure 6b. The cost function Δ*z* takes the global optimal value at the black circle in Figure 6b. At this point, the expressions of *L* and *N* are:

(43) $$L=\left[\frac{y(\sigma_{vm})}{2}\right]=\left[{\left(\frac{3\lambda^{2}}{32\pi^{4}\tau^{4}\sigma_{vm}^{2}(E/E_{n})}\right)}^{1/3}\right],N=2$$

where the square brackets indicate rounding. Equation (43) shows that when *y*(*σ_v_*_m_) < 2*L*_mid_, the layer thickness can be minimized by first setting the number of repeats to 2 and then determining code length by the velocity estimation accuracy constraint. The minimum layer thickness Δ*z*_min_ obtained in this case is:

(44) $$\Delta z_{\min}=\frac{c}{2}L\tau =\frac{c}{4}{\left(\frac{3\lambda^{2}}{4\pi^{4}\tau \sigma_{vm}^{2}(E/E_{n})}\right)}^{1/3}$$

In summary, the longest code length and the smallest number of repeats in the feasible region can minimize the layer thickness in the WSAP method.

## 4. Experiments and Verification

An experimental platform based on an onshore calibration system for ADCP is built to verify the effectiveness of the proposed waveform design methods.

### 4.1. Overview of Experimental Platform

**Figure 7:**
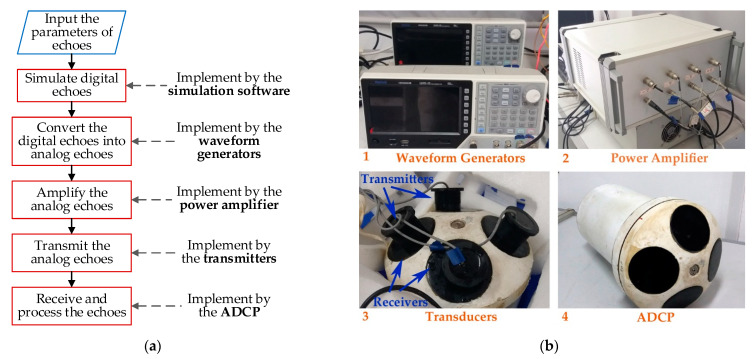
The experimental platform based on an onshore calibration system for ADCP: (**a**) workflow chart of the platform; (**b**) photos of the platform components.

The onshore calibration system can generate multi-channel simulated current measurement echo according to the set parameters and simulate underwater acoustic current measurement through seamless docking with the transducers of current measurement instruments in the laboratory. The onshore calibration system can flexibly set the transmitted waveform (including noncoherent pulse, coherent pulses train and phase-encoded pulse), water depth, scatterer distribution, current distribution, environmental noise etc., which provides convenience for the study of acoustic current measurement methods and the test of current measurement instruments. The shore calibration system consists of a computer (including acoustic Doppler echo simulation software), an arbitrary wave generator, a power amplifier, a transducer and a current measuring instrument (taking the 600 kHz ADCP as an example). The workflow chart of the onshore calibration system is shown in Figure 7a. The echo simulation software running on the computer is responsible for generating the digital echoes according to the set parameters. The screenshot of the software interface is shown in the upper left corner of Figure 7b. The arbitrary waveform generator A and B convert the digital echoes into analog signals, and their actual photos are shown in the upper right corner of Figure 7b. Each arbitrary waveform generator can generate two channels of analog signals at the same time. The power amplifier is responsible for amplifying the analog signals to an appropriate power level and matching appropriate interface impedances for all the transducers. The photo of the power amplifier is shown in the lower right corner of Figure 7b. Finally, four transducers connected the output of the power amplifier are docked with transducers of an ADCP one by one. The photo of four groups of connected transducers is shown in the lower left corner of Figure 7b. The main parameters of the experimental platform are shown in Table 1.

### 4.2. VEAP Waveform Optimization Experiment Result

This experiment is aimed to compare the velocity estimation performance of the waveform obtained by the proposed VEAP method and other waveforms under the constraints of stratification accuracy requirement and current velocity range. This experiment contains two parts: a low velocity case and a high velocity case.

#### 4.2.1. Low Velocity Case

The onshore calibration system is set as follows: the current velocity of all layers is set to 0.37 m/s, so the maximum current velocity *v*_m_ equals 0.37 m/s, and the maximum allowable layer thickness Δ*z*_m_ is set to 0.8 m. The default code is Barker code, and the MPS code is used when there is no corresponding length Barker code. The four codes selected in this experiment are shown in Table 2 [33].

Three waveforms which are different from the waveform obtained by the VEAP method in this paper are selected, and the standard deviation of velocity estimation of the four waveforms under eight *SNR* (5–40 dB) is compared. Under each *SNR* condition, the sample number of velocity estimation is 500. The process of calculating the waveform parameter of VEAP method is described as follows: the chip width is 20 μs obtained from the system bandwidth, and the chip width is fixed. According to the value of Δ*z*_m_ and *v*_m_, this measurement application belongs to the case of *v*_m_Δ*z*_m_ ≥ *cλ*/16. According to Equation (28), the code length and number of repeats that minimize the velocity standard deviation are *L* = 42 and *N* = 2. At the same time, the correlation delay is *t*_d_ = 0.84 ms, the average time of phase difference is *t*_a_ = 0.84 ms and the layer thickness is Δ*z* = 0.63 m. In addition, other three waveforms are selected in the feasible region, and their waveform parameters are (*N* = 3, *L* = 23), (*N* = 5, *L* = 13) and (*N* = 8, *L* = 7).

**Figure 8:**
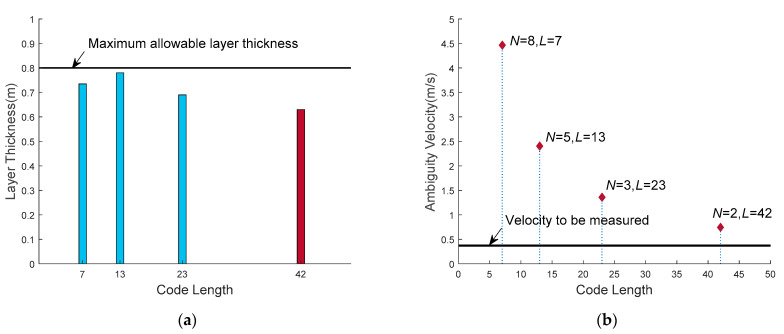
The layer thickness and velocity estimation range of the four waveforms all meet the constraint conditions: (**a**) layer thickness achieved by four waveforms; (**b**) velocity estimation range achieved by four waveforms.

The values of layer thickness achieved by the four waveforms are shown in Figure 8a. They are 0.73 m, 0.78 m, 0.69 m and 0.63 m, which are all smaller than Δ*z*_m_, and all meet the stratification accuracy constraint. The velocity estimation range achieved by the four waveform is shown in Figure 8b, respectively 4.5 m/s, 2.4 m/s, 1.4 m/s and 0.74 m/s, which are all greater than two times of the *v*_m_, and all meet the current velocity range constraint.

**Figure 9:**
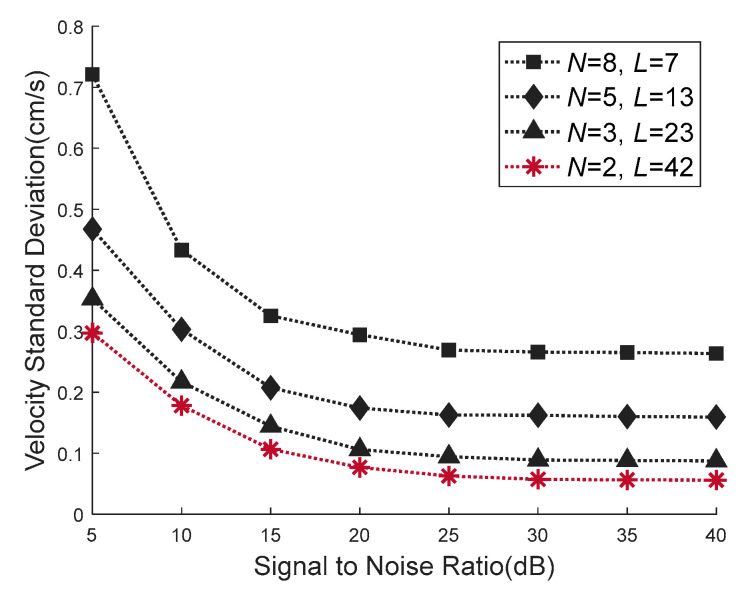
Comparison of the velocity standard deviation between four waveforms. The waveform (*N* = 2, *L* = 42) obtained by the proposed VEAP method achieves the smallest velocity standard deviation.

The standard deviation of velocity estimation realized by the four waveforms is shown in Figure 9. The dotted line with star icon indicates the velocity standard deviation of the waveform (*N* = 2, *L* = 42) obtained by the VEAP method in this paper. The triangle icon indicates the deviation of (*N* = 3, *L* = 23) waveform. The diamond icon indicates the deviation of (*N* = 5, *L* = 13) waveform. The square icon indicates the deviation of (*N* = 8, *L* = 7) waveform. It can be seen from Figure 9 that the velocity standard deviation of four waveforms decreases with the increase of *SNR*. When *SNR* is greater than 25 dB, the velocity standard deviation tends to be stable, and the stable values ordered from largest to smallest are 0.22 cm/s, 0.14 cm/s, 0.1 cm/s and 0.05 cm/s. Under all *SNR* conditions, the velocity estimation accuracy of the waveform designed by the proposed VEAP method is better than that of the other waveforms, which proves the effectiveness of the proposed method.

#### 4.2.2. High Velocity Case

The onshore calibration system is set as follows: the current velocity of all layers is set to 2 m/s, so the maximum current velocity *v*_m_ equals 2 m/s, and the maximum allowable layer thickness Δ*z*_m_ is set to 0.8 m. According to Equation (28), the code length and number of repeats that minimize the velocity standard deviation are *N* = 8 and *L* = 7. In addition, other three waveforms are selected in the feasible region, and their waveform parameters are (*N* = 9, *L* = 6), (*N* = 10, *L* = 5) and (*N* = 12, *L* = 4).

**Figure 10:**
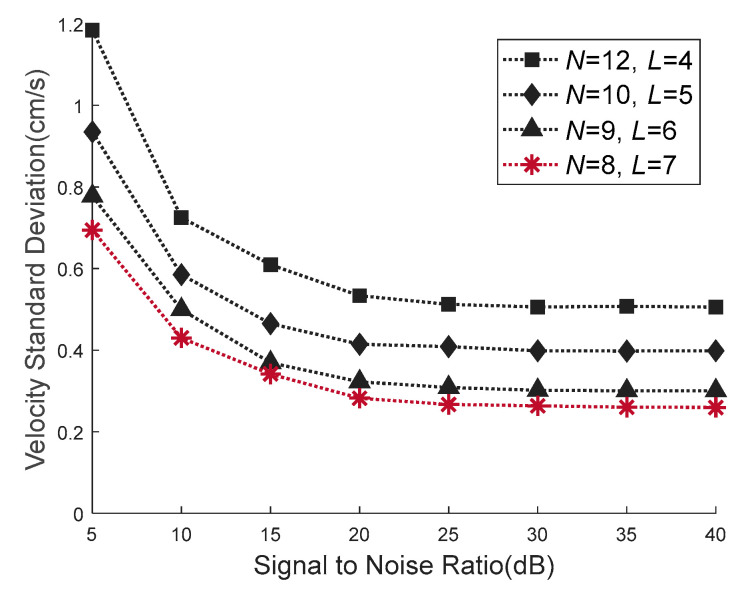
Comparison of the velocity standard deviation between four waveforms. The waveform (*N* = 8, *L* = 7) obtained by the proposed VEAP method achieves the smallest velocity standard deviation.

The standard deviation of velocity estimation realized by the four waveforms is shown in Figure 10. The dotted line with star icon indicates the velocity standard deviation of the waveform (*N* = 8, *L* = 7) obtained by the proposed VEAP method. The triangle icon indicates the deviation of (*N* = 9, *L* = 6) waveform. The diamond icon indicates the deviation of (*N* = 10, *L* = 5) waveform. The square icon indicates the deviation of (*N* = 12, *L* = 4) waveform. Under all SNR conditions, the velocity estimation accuracy of the waveform designed by the proposed VEAP method is better than that of the other waveforms, which proves the effectiveness of the proposed method.

### 4.3. WSAP Waveform Optimization Experiment Result

This experiment aimed to compare the stratification performance of the waveform obtained by the proposed WSAP method and other waveforms under the constraints of velocity estimation accuracy requirement and current velocity range. The onshore calibration system is set as follows: In order to simulate shear current application, the current velocity is set to be linearly distributed with depth. The velocity of top layer is 1 m/s, and the velocity of bottom layer is 0.1 m/s, so the maximum current velocity to be measured is *v*_m_ = 1 m/s. The maximum allowable velocity standard deviation is *σ_v_*_m_ = 0.05 m/s. The default code is Barker code, and the MPS code is used when there is no corresponding length Barker code. The four codes selected in this experiment are shown in Table 3 [33].

Three waveforms that are different from the waveform obtained by the WSAP method in this paper are selected, and the layer thickness of the four waveforms under 20 dB *SNR* is compared. The sample number of velocity estimation is 500. The process of calculating the waveform parameter of WSAP method is described as follows: The chip width is 20 μs obtained from the system bandwidth. *y*(*σ_v_*_m_) = 16.8 is obtained from the maximum allowable velocity estimation error *σ_v_*_m_, and *L*_mid_ = 15.6 is obtained from the maximum current velocity *v*_m_. Therefore, this measurement application belongs to the case of *y*(*σ_v_*_m_) < 2*L*_mid_. According to Equation (43), the code length and number of repeats that minimize the layer thickness is *N* = 2 and *L* = 9. At the same time, the correlation delay is *t*_d_ = 0.18 ms and the average time of phase difference is *t*_a_ = 0.18 ms. In addition, the other three waveforms are selected in the feasible region, and their waveform parameters are (*N* = 3, *L* = 6), (*N* = 4, *L* = 5) and (*N* = 5, *L* = 4).

**Figure 11:**
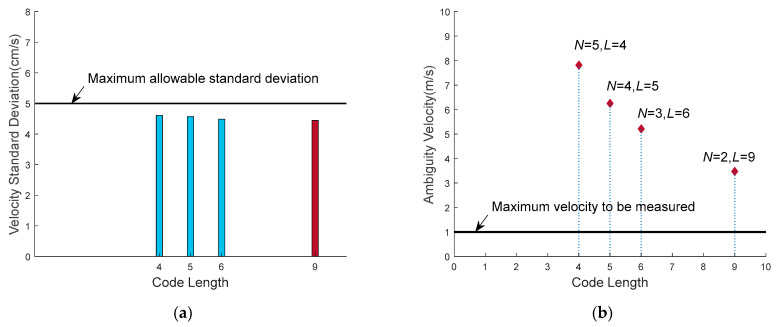
Velocity standard deviation and velocity estimation range of the four waveforms all meet the constraint conditions: (**a**) velocity standard deviation achieved by four waveforms; (**b**) velocity estimation range achieved by four waveforms.

The values of velocity standard deviation achieved by the four waveforms are shown in Figure 11a. They are 4.58 cm/s, 4.77 cm/s, 4.61 cm/s and 4.45 cm/s, which are all smaller than *σ_v_*_m_, and all of them meet the velocity estimation accuracy constraint. The velocity estimation range achieved by the four waveform is shown in Figure 11b, respectively 7.8 m/s, 6.3 m/s, 5.2 m/s and 3.5 m/s, which are all greater than two times of the *v*_m_, and all meet the current velocity range constraint.

**Figure 12:**
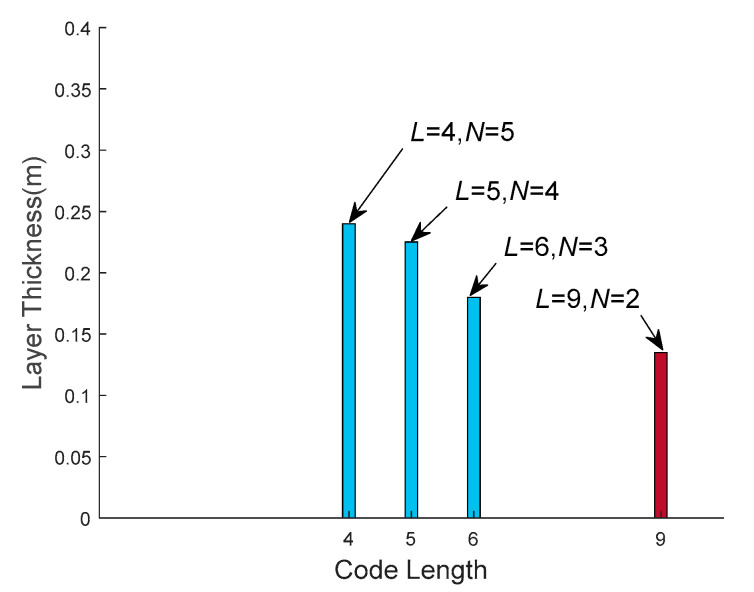
Comparison of the layer thickness between the waveform obtained by the proposed WSAP method and the other waveforms. The waveform (*N* = 2, *L* = 9) obtained by the proposed WSAP method achieves the smallest layer thickness.

The layer thickness obtained by the four waveforms is shown in Figure 12. The black vertical bar indicates the layer thickness of the waveform (*N* = 2, *L* = 9) obtained by the WSAP method in this paper. The gray vertical bars indicate the layer thickness of the other waveforms (*N* = 3, *L* = 6), (*N* = 4, *L* = 5) and (*N* = 5, *L* = 4), respectively. It can be seen from Figure 12 that the layer thickness of the four waveforms decrease from left to right, and the values are 0.24 m, 0.23 m, 0.18 m and 0.14 m, respectively. The stratification accuracy of the waveform designed by the proposed WSAP method is better than that of the other waveforms, which proves the effectiveness of the proposed method.

## 5. Conclusions

In order to overcome the shortcomings of the existing waveform design methods, such as lack of application environment pertinence and difficulties in optimizing the estimation performance, this paper designs two waveform optimization methods with environmental adaptability and optimal estimation performance. In these two methods, the environment characteristics such as current velocity range and distribution are taken as the constraints of the nonlinear optimization problems to realize the velocity estimation or stratification performance optimization.

The proposed methods improve the velocity estimation accuracy, stratification accuracy and environmental adaptability of broadband ADCP. Evaluation of algorithm performance in comparison with other existing methods indicate that the velocity estimation accuracy of the proposed VEAP method is improved by at least 50%, and the stratification accuracy of the proposed WSAP method is improved by at least 22%, which demonstrates the proposed methods are accurate and effective.

Obtaining high-precision velocity estimation and water stratification is the primary purpose of most ADCP measurement applications, so the proposed methods in this paper are valuable for many measurement activities. The waveform parameters can be dynamically configured according to the characteristics of the measurement environment by using the proposed methods, then higher accuracy and flexibility can be obtained than the fixed parameter measurement method.

However, there may be some limitations in practical applications. For example, it is sometimes not easy to obtain accurate characteristics of measurement environment. The environmental characteristics obtained by experience and rough estimation sometimes have certain errors. Therefore, research on obtaining accurate environmental characteristics will be interesting and valuable.

## Figures and Tables

**Table 1:** The parameters of the experimental platform.

	Parameters	Value
1	Operating Frequency	600 kHz
2	System Bandwidth	50 kHz
3	Minimum Layer Thickness	0.1 m
4	Velocity Resolution	0.5 mm/s
5	Beam Width	3 degrees
6	Beam Inclination	30 degrees
7	Velocity Range	±20 m/s
8	Maximum Profile Range	60 m

**Table 2:** Codes used in the experiment of VEAP waveform design.

Code Length	Code Type	Hexadecimal	Binary
7	Barker	0x32	1110010
13	Barker	0x1F35	1111100110101
23	MPS	0x38FD49	01110001111110101001001
42	MPS	0x4447B874B4	000100010001000111101 110000111010010110100

**Table 3:** Codes used in the experiment of WSAP waveform design.

Code Length	Code Type	Hexadecimal	Binary
4	Barker	0xD	1101
5	Barker	0x1D	11101
6	MPS	0x34	110100
9	MPS	0xD7	011010111

## Data Availability

Not applicable.
